# Unsupervised Port Berth Localization from Automatic Identification System Data

**DOI:** 10.3390/s25226845

**Published:** 2025-11-08

**Authors:** Andreas Hadjipieris, Neofytos Dimitriou, Ognjen Arandjelović

**Affiliations:** 1 Cyprus Marine and Maritime Institute, Vasileos Pavlou Square 13, Larnaca 6023, Cyprus; antreashp@gmail.com (A.H.); neofytosd@gmail.com (N.D.); 2 School of Computer Science, University of St Andrews, St Andrews KY16 9AJ, UK

**Keywords:** maritime, shipping, AIS, spatial clustering, machine learning, minimum description length, hyperparameter tuning

## Abstract

Port berthing sites are regions of high interest for monitoring and optimizing port operations. Data sourced from the Automatic Identification System (AIS) can be superimposed on berths, enabling their real-time monitoring and revealing long-term utilization patterns. Ultimately, insights from multiple berths can uncover bottlenecks, and lead to the optimization of the underlying supply chain of the port and beyond. However, publicly available documentation of port berths, even when available, is frequently incomplete—e.g., there may be missing berths or inaccuracies such as incorrect boundary boxes—necessitating a more robust, data-driven approach to port berth localization. In this context, we propose an unsupervised spatial modeling method that leverages AIS data clustering and hyperparameter optimization to localize berthing sites. Trained on one month of freely available AIS data and evaluated across ports of varying sizes, our models significantly outperform competing methods, achieving a mean Bhattacharyya distance of 0.85 when comparing Gaussian Mixture Models trained on separate data splits, compared to 13.56 for the best existing method. Qualitative comparison with satellite images and existing berth labels further supports the superiority of our method, revealing more precise berth boundaries and improved spatial resolution across diverse port environments.

## 1. Introduction

The Automatic Identification System (AIS) plays a pivotal role in the digitization of the shipping industry by providing frequent vessel movement data [1]. An AIS transponder is mandatory for all ships with a gross tonnage over 300 that sail in international waters, ships over 500 tons that do not sail internationally, and all passenger ships [2]. With over 310 billion AIS messages transmitted every year, the maritime sector has unquestionably entered the big data era [3]. Originally intended to prevent ship collisions [1], ongoing improvements in data quality and coverage have greatly expanded the potential applications of AIS data. However, despite accessibility to free AIS data with the appropriate infrastructure (e.g., base stations), organizations that collect and store global AIS data typically charge for access, creating a significant barrier to entry, and hindering the big data potential in the maritime sector. Recently, a few providers, most notably AISStream (https://aisstream.io/ accessed on 4 November 2025) and AISHub (https://aishub.net accessed on 4 November 2025), have started offering real-time terrestrial AIS data at no cost. In this work, we focus on open-access sources, which can be sustainably collected across both large spatial and temporal scales. To address the challenge of incomplete port berth documentation, we propose an unsupervised spatial modeling framework that leverages freely available AIS data to localize berthing sites.

**Figure 1:**
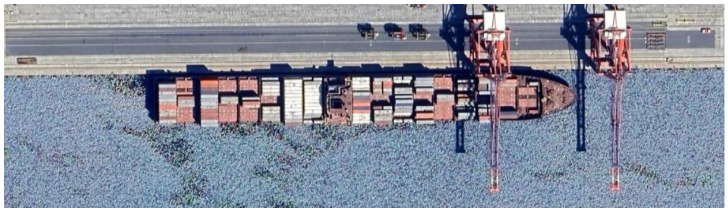
Port berth in Limassol. Satellite image from Google Maps [4] showing a typical berth used for loading and unloading container ships in Limassol Port.

Port berths are designated locations within ports equipped with necessary facilities such as cranes and docking infrastructure (see Figure 1) that support essential maritime operations including cargo loading and unloading, refueling, and maintenance. Despite their critical role, many ports fail to document their location adequately, and existing documentation is often fraught with inaccuracies or omissions.

By accurately identifying and localizing port berths, we aim to fill these data gaps, ensuring that such vital information is both freely available and reliable. Importantly, port berth localization can enable AIS geofencing, i.e., AIS data overlaid on a set of berths to discern short-term and long-term patterns. Examples of applications include the generation of real-time and historical statistics, e.g., service time, the development of predictive data-driven models, and the use of data, statistics, and models by port authorities to make informed decisions regarding infrastructure investments, expansions (e.g., new terminals), or operational adjustments [5]. The shortage of complete and accurate port berth documentation motivates the development of automated berth localization methods. However, this problem remains challenging for several reasons: the inherent absence of reliable ground truth labels necessitates a fully unsupervised approach; AIS data are noisy, making it difficult to reliably distinguish stopped vessels from transient behaviours beyond simple speed-based criteria; accurately detecting port berths requires identifying collective spatial–temporal vessel patterns rather than single-vessel movements, inherently calling for hierarchical clustering approaches; and existing clustering methods typically demand extensive parameter fine-tuning, limiting their generalizability across diverse ports. Collectively, these factors underscore the complexity of developing robust, fully unsupervised, and universally applicable berth localization methods. However, existing research addressing this problem remains scarce, as discussed in Section 1.

In this work, we devise an unsupervised method for optimizing data-driven models [6] and demonstrate its efficacy across various ports around the globe. By selecting a diverse set of ports (based on TEU), we ensure our sample encapsulates a wide range of port sizes and operational contexts, from small sea ports to some of the world’s largest shipping hubs (see Table 1). The promising results of our method across 11 ports underscore the proposed method’s adaptability and its potential applicability to ports globally. Our contributions are as follows:

- We propose a data augmentation strategy based on spatial information that is readily available from AIS data, and experimentally validate its efficacy. This augmentation increases the information regarding the occupying space of a vessel by considering its dimensions and heading.
- We introduce a score based on Kullback–Leibler divergence (KLD) [7,8] and a hyperparameter optimization evaluation approach and utilize them to explore model selection based on a novel combination of Bayesian optimization and the minimum description length (MDL) [9,10].
- We utilize a post-processing step to translate the underlying distributions of Gaussian Mixture Models (GMMs) [11] to polygons of predicted port berths, and conduct a comparative analysis on port berth localization using as a metric the Bhattacharyya distance (BD) [12,13] based on Monte Carlo Integration (MCI).

### Related Work

**Vessel Stop Detection and Port Area Clustering.** Identifying vessel stop episodes and berth locations from AIS data involves detecting meaningful spatial–temporal patterns. Wang and McArthur [14] and Nogueira et al. [15] exploited temporal and spatial gaps in GPS data to detect stop episodes and distinct movement patterns. Density-based methods, particularly DBSCAN, have proven effective in pinpointing stay points, as demonstrated by Hwang et al. [16] and Luo et al. [17]. Nevertheless, conventional DBSCAN faces scalability limitations, prompting advancements such as GriT-DBSCAN by Huang et al. [18], which introduced efficient grid-tree indexing, and the variant by Chen et al. [19], optimized for high-dimensional data by pruning unnecessary computations. Adaptive trajectory clustering methods were introduced by Tang et al. [20], who employed Douglas–Peucker and OPTICS algorithms, while Wang et al. [21] enhanced sequential trajectory clustering through structured pattern representations.

Spatial clustering methods explicitly tailored for maritime contexts have also been explored. Millefiori et al. [22] adapted Kernel Density Estimation within a map-reduce framework for defining port boundaries. Xiao et al. [23] combined DBSCAN and GMMs to spatially cluster urban areas, and Yan et al. [24] integrated DBSCAN with Random Forests to identify vessel stopping information. The dynamic clustering method by Rehman and Belhaouari [25] avoided predefined cluster counts by dynamically adjusting granularity through iterative splitting and merging. Steenari et al. [26] specifically applied DBSCAN to berth localization, providing a useful comparative framework.

**Unsupervised and Parameter-Free Clustering Methods.** Acknowledging the challenges of parameter tuning, recent methodologies emphasize fully unsupervised, parameter-free clustering. Ansari et al. [27] provided an extensive review of such unsupervised approaches, including DBSCAN, OPTICS, and GMMs. End-to-end frameworks such as Neural Mixture Models with Expectation-Maximization by Tissera et al. [28], and Minimum Description Length-based spatial clustering by Kirkley [29], represent significant advances toward automated clustering without manual intervention. Additionally, Zhang et al. [30] utilized reinforcement learning to automate hyperparameter optimization in DBSCAN, reinforcing the potential of fully autonomous clustering methods.

**Internal Validation Metrics for Unsupervised Clustering.** Due to the absence of ground truth in berth localization, internal validation metrics are crucial. Chen et al. [31] and Wang et al. [32] employed KLD to internally refine clustering outcomes. Punera and Ghosh [33] further validated KLD in consensus clustering frameworks, ensuring robust cluster structures from internal data distributions. Similarly, BD was effectively applied by Lahlimi et al. [34] in hyperspectral band selection, and by You et al. [35] in speaker recognition, demonstrating the reliable unsupervised validation of clustering consistency.

Collectively, these works underscore the non-triviality of developing robust unsupervised, data-driven clustering methods, particularly in complex spatial–temporal contexts where ground truth is absent. Despite significant methodological advances, a application-specific method that is explicitly tailored and rigorously validated for port berth localization across ports of varying sizes and characteristics remains lacking.

The rest of the paper is organized as follows. In Section 2, we detail our method, including data preprocessing steps, spatial data augmentation strategy, model selection, and the use of GMMs for spatial clustering. Our experimental findings are detailed in Section 3. We conclude in Section 4, where we discuss our findings and contextualize them within the broader scope of port optimization.

## 2. Materials and Methods

### 2.1. AIS Data

**Figure 2:**
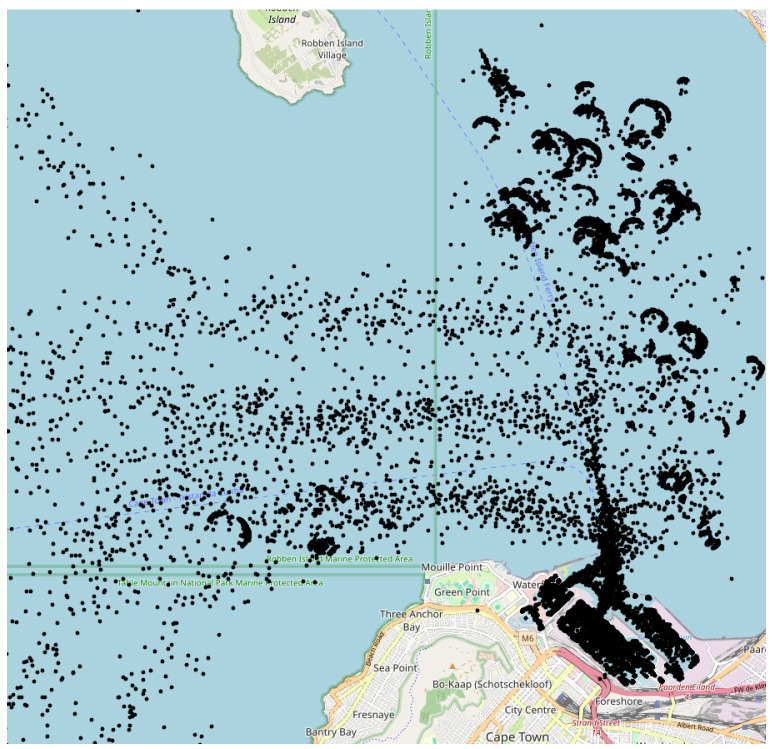
AIS-based traffic for a month—Cape Town. Automatic Identification System (AIS) position reports (black dots) recorded during one month for the port of Cape Town and its surrounding waters.

**Figure 3:**
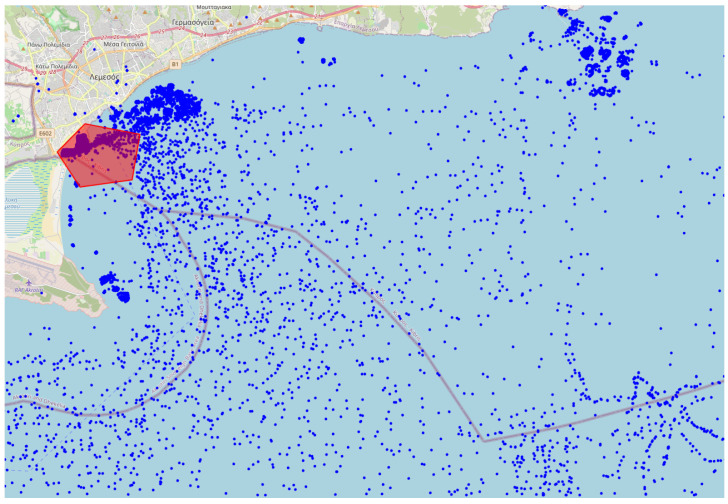
AIS-based traffic for a month—Limassol. Automatic Identification System (AIS) position reports (blue dots) collected over one month for Limassol port (red pentagon marks the port’s area).

The AIS data utilized in this research were sourced using the Application Programming Interface (API) of AISStream.io, a platform that offers free access to terrestrial AIS data. Their network of AIS receiving stations, which varies in density globally, covers areas within approximately 200 km of the majority of the world’s coastlines. For a detailed visualization, a map of their AIS stations is available on their website. To receive AIS data from AISStream.io, one needs to define the region or regions of interest (ROIs) and, optionally, a list of vessels of interest based on Maritime Mobile Service Identities (MMSIs). The MMSI is a nine-digit number used for identifying vessels, ship stations, and coast stations in maritime communication and navigation systems. However, while an MMSI is linked to a single ship at a given time, it can be reassigned to different ships over time, and a ship might change its MMSI. Therefore, the MMSI is not a unique identifier, though it is used as an indicator for individual ships in this work. Finally, we do not utilize the vessel-specific filtering of the API. AIS data from the Cape Town and Limassol ports over a period of one month are shown in Figure 2 and Figure 3, respectively. The port-specific ROI is an area covering an entire port while avoiding adjacent waterways or nearby ports. An example of the Limassol ROI compared to all the data collected for that port is shown in Figure 3. To ensure a representative sample of ports of all sizes (based on TEU) the following sampling technique was employed. First, twelve port sizes in the range of 100 to 50,000 TEU were sampled using a uniform logarithmic distribution. Ports corresponding closely to these predetermined size categories were then selected (see Table 1).

#### 2.1.1. Period of Interest

We fixed the maximum period of interest (POI) at one month (1–31 October 2023), and collected AIS data across the ROIs. This setup reflects our scope: identifying port berths given up to one month of freely available AIS data. For the sensitivity analysis of the amount of data (discussed in Section 3), the POI varied. Specifically, we carried out four experiments with POIs that begin on the 1st of October and span three days, one week, two weeks, and the entire month, respectively.

The number of AIS messages received over a period of 1 month for each port is presented in Table 1 alongside each port’s TEU throughput. Almost no AIS messages were received for the port of Ambarli, which was therefore excluded.

#### 2.1.2. Boundary Boxes for Port Berths

**Figure 4:**
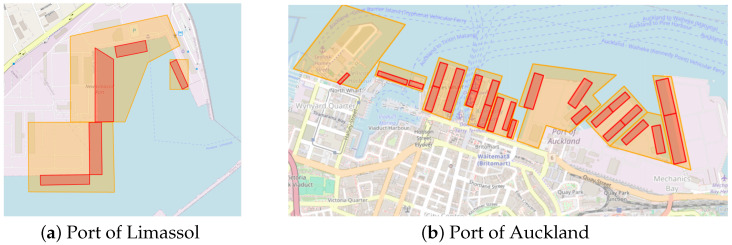
ShipNext berth and terminal labels. Examples from Limassol (**a**) and Auckland (**b**); red rotated rectangles denote berth annotations, and orange polygons denote terminal areas.

For qualitative model evaluation, rather than training, we collected port berth boundary boxes for the ports of interest. In particular, berth, terminal, and port area labels were obtained from the ShipNext website (https://shipnext.com/ accessed on 4 November 2025). The port berth areas are represented as rotated rectangles, as illustrated in Figure 4.

### 2.2. Methodology

#### 2.2.1. Data Preprocessing

The preprocessing of the raw AIS data involved data cleaning, interpolation, and standardisation. For data cleaning, the data are filtered based on vessel type, so that only cargo and tanker ships remain, and speed (<3 knots). Second, AIS records with either incomplete vessel dimensions or missing heading values are removed. Finally, for each vessel, consecutive messages indicating a substantial directional change in the heading are removed (>10 degrees), since large directional changes may be due to vessel movement or due to noisy or incorrect recorded values.

Following the above, the AIS messages per MMSI are resampled such that there is at maximum one AIS message per hour (“interpolation” step). This is achieved by taking the last message of every hour, if available, for each vessel. An ablation study is carried out to determine the interpolation period, as discussed in Section 3.2. Finally, the latitude and longitude information of all AIS messages are standardized to have a mean of 0 and a standard deviation (SD) of 1.

#### 2.2.2. Data Split

For the purposes of hyperparameter tuning and evaluation (but not inference, see Section 2.4), the preprocessed AIS data for each port was split into two datasets. The splitting process involves the following steps:

- Vessels are sorted in descending order based on the number of AIS messages they transmitted.
- The sorted vessels are separated into two data sets by alternately assigning consecutive vessels to a different data set.

This methodology ensures that the resulting splits of the data have approximately equal numbers of AIS messages while maintaining vessel exclusivity within each split.

#### 2.2.3. DBSCAN

Density-Based Spatial Clustering of Applications with Noise (DBSCAN), first introduced by Ester et al. [36], is a non-parametric, density-based clustering algorithm, which groups points in a data set based on their proximity and the density of their surrounding points. DBSCAN is governed by two hyperparameters: ϵ which defines the maximum radius of a neighborhood, and $p_{n}$ which refers to the minimum number of points for a cluster to be formed.

**Figure 5:**
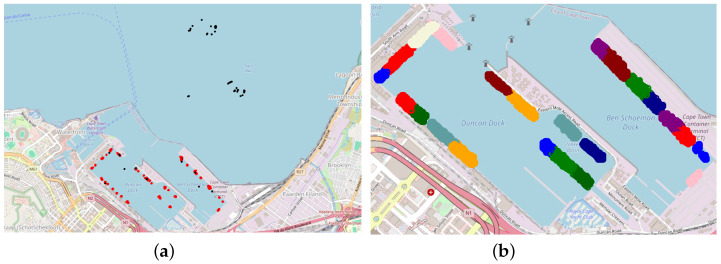
(**a**) DBSCAN retains in-cluster AIS points (red) and flags outliers (black). (**b**) Components (±3 SDs) of optimal hyper-parameter GMM represents candidate berths.

In the present work, DBSCAN is applied to each vessel independently, clustering AIS data points based on vessel movement (or lack thereof). Since the AIS data of each vessel are reported hourly, intuitively, the algorithm creates a cluster in locations where the vessel reportedly stayed for at least $p_{n}$ hours, as reported by AIS messages, with each reported location being within a distance of ϵ from one another. Note that haversine distance was used to account for differences in distances between points at different latitudes. DBSCAN effectively identifies and filters out outliers—in our case, these can be AIS messages with vessel movement or insufficient duration of stay. Such points are shown as black dots in Figure 5a. We optimize these two hyperparameters using the Tree Parzen Estimator (TPE), with a log uniform distribution of real numbers between 5 and 70 as the prior for ϵ, and a uniform distribution of integers between 2 and 25 as the prior for $p_{n}$.

#### 2.2.4. Spatial Data Augmentation

**Figure 6:**
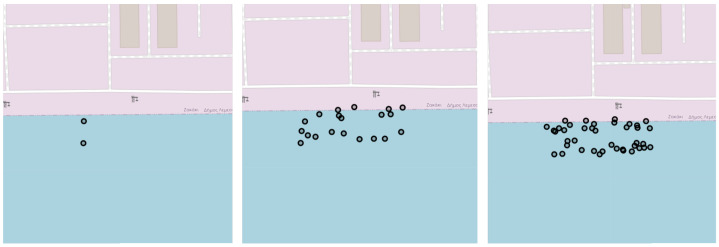
Proposed spatial data augmentation mechanism. Starting from two AIS messages (**left**), 10 synthetic points (**centre**) and 20 synthetic points (**right**) are generated per message.

Following DBSCAN-based filtering, for each remaining AIS message, we generate additional points (10 during training, 20 during evaluation) by sampling from a uniform distribution within the area occupied by the vessel. This area is determined based on the vessel’s dimensions and heading, which are provided in the AIS data. The dimensions of the vessel are captured in four fields: “Dimension A”, “Dimension B”, “Dimension C”, and “Dimension D”. These fields report the distances of the AIS receiver to each of the ship’s four sides. Based on these dimensions and the reported heading of the vessel, further points are generated as shown in Figure 6.

#### 2.2.5. Geohash Encoding

Following the above, the spatial data of the AIS messages (i.e., longitude, latitude) can be encoded as geohashes—a practice that aims to declutter the dataset while significantly improving the efficiency of model training and inference. Guided by domain knowledge on cargo/tanker berth sizes and typical berth spacing, we set geohash precision to 9, which bins the data into 4.8 m × 4.8 m squares, to declutter AIS points without limiting spatial resolution. We report on the effectiveness of our proposed method with and without geohash encoding in Section 3.

#### 2.2.6. Gaussian Mixture Model

A GMM is a probabilistic model that represents the data as a mixture of several Gaussian distributions, each of which can be thought of as a cluster [11]. GMM does not assign data points to single clusters though; instead, it assigns a probability distribution across all Gaussian components for each data point. One important hyperparameter in GMMs is the number of components (“ncomponents”), which dictates the number of Gaussian distributions underlying the model. In our context, the Gaussian distributions will be interpreted as representing potential berths in a port (illustrated in Figure 5b). Therefore, the value of “ncomponents” determines how many distinct berths (or other docking areas) a GMM will attempt to model. To determine the optimal “ncomponents”, we use the Minimum Description Length (MDL) criterion [9], which can be viewed as a formalization of Occam’s razor [10]. It provides a principled approach of balancing model complexity against explanatory power in light of the observed data [10]. Succinctly, MDL can be viewed as an approximation to Bayesian model selection, but without the requirement of an explicit prior. Instead of assigning subjective priors, it uses universal coding principles to measure model complexity objectively. Here, the description length is the length, in bits, needed to encode both the parameters of a GMM, and the data given the GMM [10]. Formally:

(1) DL(M,D)=L(M)+L(D|M)

where DL(M,D) is the description length corresponding to the model *M* and data *D* (given *M*) respectively. The description length can be further elaborated as follows:

(2) $$DL\left(M,D\right)=\frac{1}{2}N_{M}\log_{2}N-\sum_{i=0}^{N-1}\log_{2}P\left(d_{i}\right)$$

where $d_{i}$ are individual data points from *D*, *N* their count, and $N_{M}$ the number of GMM parameters [10]. The number of parameter $N_{M}$ of a GMM with $N_{C}$ “ncomponents” can be written as follows:

(3) $$N_{M}=N_{C}\times \left(2\times N_{F}+\frac{N_{F}^{2}-N_{F}}{2}+1\right)-1$$

Here, $N_{C}$ is the number of mixture components, i.e., number of Gaussians in the GMM, and $N_{F}$ is the number of dimensions of the (input) data (in our case, it is 2). Intuitively, Equation (3) accounts for the mean, covariance matrix, and weight parameters that come with every Gaussian.

This MDL-based approach to tuning “ncomponents” balances model complexity with goodness of fit, helping to avoid underfitting (too few berths modeled) or overfitting (e.g., modeling noise as berths). The process is as follows:

- We define a range of possible “ncomponents” values: (3, 50) for smaller ports and (30, 250) for larger ports (Singapore and Antwerp in our case).
- We fit a GMM with full covariance matrix, 2 restarts, and $1\times 10^{-4}$ tolerance for each “ncomponents” value and for each data split.
- We calculate the MDL for each fitted GMM on each data split. The “ncomponents” value yielding the lowest average MDL across the two data splits is selected.

#### 2.2.7. Hyperparameter Tuning Based on Kullback–Leibler Divergence

The following summarizes our hyperparameter optimization process:

- We use the Tree-structured Parzen Estimator (TPE) to search the space of DBSCAN’s hyperparameters (ϵ and $p_{n}$) for 100 trials, with the first 30 serving as a warm start.
- At every trial, we fit two GMMs, one for each augmented, as previously defined, data split, using the optimal “ncomponents” value (determined as discussed in Section 2.2.6).
- We assess the similarity between the two GMMs using the symmetric KLD, as described in the equations that follow.
- Following the results on 100 trials, the trial yielding the lowest KLD score is selected as the best configuration (i.e., optimal ϵ and $p_{n}$ for DBSCAN and optimal “ncomponents” based on the MDL criterion) for that port.

We use Optuna for hyperparameter optimization [37]. The KLD [7] from distribution *p* to distribution *q* is defined as follows:

(4) $$KL\left(p||q\right)=\int p\left(x\right)\log\frac{p(x)}{q(x)}dx$$

To ensure symmetry in our comparison, we use a symmetric variant of KLD which we define as:

(5) $$KL_{symm}\left(p,q\right)=\max(KL(p||q),KL(q||p))$$

where *p* and *q* in our case represent the underlying distribution of GMMs fitted on different data splits.

The symmetric KLD quantifies the similarity between GMMs trained on different data splits, reflecting how well the chosen hyperparameters (of both DBSCAN and GMM) generalize across these complementary subsets of the data. Intuitively, a lower KLD indicates that the GMMs trained on different data splits identify a similar set of berths, suggesting that the model’s representation of port structure is consistent and robust across subsets of the data. The optimal hyperparameter configurations for all ports, automatically selected based on the described process, are listed in Appendix B.

### 2.3. Evaluation Approach

During evaluation, to assess the consistency and robustness of our models across different data splits, we employ the BD rather than the KLD. For hyperparameter tuning, KLD was more suitable as it more heavily penalizes inconsistencies. In contrast, for evaluation, the BD provides a more balanced assessment (especially since true ground truth is lacking), measuring distribution similarity without overly penalizing minor variations between GMMs trained on different data splits. The evaluation process includes the following steps:

- Train GMMs on each of the two augmented data splits (generating 20 points rather than 10) using tuned hyperparameters for both DBSCAN and GMM. GMM hyperparameters “tolerance” and number of restarts are set to $1\times 10^{-5}$ and 5 respectively.
- Uniformly sample points from the port area.
- Calculate the probability of these points under each of the two GMMs.
- Use these probabilities to compute the Bhattacharyya coefficient.

The Bhattacharyya coefficient is defined as follows:

(6) $$BC\left(P,Q\right)=\int_{x}\sqrt{p(x)q(x)}dx$$

where p(x) and q(x) are the values of the probability density functions (PDFs) evaluated at the point x for the two GMMs respectively. To estimate this coefficient over the entire port area, we use Monte Carlo Integration with a uniform distribution:

(7) $$BC_{MCI}\left(P,Q\right)=A\int_{x}\sqrt{p(x)q(x)}\frac{1}{A}dx$$

where *A* is the total area of the port. Finally, we calculate the BD by taking the negative logarithm of the coefficient:

(8) $$BD_{MCI}\left(BC_{MCI}\right)=-log\left(BC_{MCI}\right)$$

Intuitively, a lower BD indicates that the two GMMs assign similar probabilities to the same areas within the port, suggesting that our model consistently identifies similar berth structures across different data splits. For the Steenari et al. method [26], which produces non-probabilistic clusters, we assign a probability of 1 if a sample point falls within a cluster and 0 otherwise, allowing for comparison with our probabilistic approach. To obtain a robust estimate of performance, we calculate the averaged BD over 200 Monte Carlo reruns.

### 2.4. Port Berth Localization

To localize port berths, the entire data set is used alongside the optimal hyperparameter configuration of both the GMM and DBScan. Moreover, during augmentation, we generate 20 points rather than 10. Finally, the hyperparameters of the GMM, “tolerance” and number of restarts, are set to $1\times 10^{-5}$ and 5 respectively. To generate boundary boxes, the augmented AIS points that fall in each component of the final GMM (of each port) are encircled by a rotated rectangle, representing the area of the localized port berth. The rotated rectangle is calculated by finding the smallest possible rectangle that can contain all points in a cluster. To achieve this, we calculate the convex hull of the cluster points and then evaluate the bounding rectangles at different angles formed by the edges of the hull. The rectangle with the smallest area is selected as the final bounding box of each Gaussian component.

### 2.5. Use of Generative AI Tools

During the preparation of this manuscript, the authors used a generative AI tool (OpenAI ChatGPT, GPT-4) to improve the clarity and phrasing of certain text passages. The tool was not used to generate original research content, study design, data, or analysis. After using the tool, the authors carefully reviewed and edited the output to ensure accuracy and compliance with the intended meaning. The final responsibility for all content rests solely with the authors.

## 3. Results

### 3.1. Comparative Analysis

In this section, we conduct a comparative analysis between the proposed method and the method by Steenari et al. [26] on port berth localization with one month of AIS data. Experiments were executed on a Windows 11 Pro workstation (AMD Ryzen 9 5950X/3.4 GHz/16-core, Nvidia RTX 4090 GPU, 64 GB RAM, 1 TB SSD). For our method, the number of AIS messages that remain after preprocessing (but before DBSCAN and augmentation) are Algeciras (2886), Antwerp (23,309), Auckland (1268), Busan (10,380), Cape Town (2563), Gdansk (8070), Limassol (403), Livorno (3226), Los Angeles (2721), Singapore (23,521), and Southampton (1762). Further information is provided in Appendix A. For the competing method, based on the preprocessing used by Steenari et al., the number of AIS messages that remain before DBSCAN are: Algeciras (7572), Antwerp (160,368), Auckland (2784), Busan (45,030), Cape Town (40,307), Gdansk (19,056), Limassol (1051), Livorno (7173), Los Angeles (5625), Singapore (59,313), and Southampton (4146). Notably, for all ports, our method is superior even though it consistently retains less than half of the amount of AIS messages compared to the competing method.

**Figure 7:**
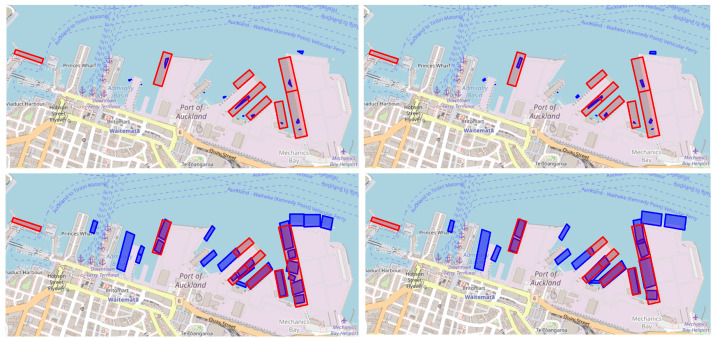
Method comparison on Auckland berths. Predicted polygons (blue) versus ShipNext labels (red) for: baseline (**top-left**), baseline with rotated rectangles (BD: 12.49) (**top-right**), proposed method without geohash encoding (BD: 1.24) (**bottom-left**), and proposed method with geohash encoding (BD: 0.92) (**bottom-right**).

The proposed method by Steenari et al. [26] begins by filtering AIS data to records within a specified port polygon (ShipNext’s port labels are used in our experiments). Next, data points with a navigational status of 5 (mooring) and a speed greater than 1 are removed. Any duplicates based on MMSI and timestamp are also removed. Continuous mooring events are subsequently identified, where each event is defined as a continuous stream of AIS messages with navigational status set to mooring, lasting more than >1 h. The AIS messages corresponding to an event are assigned a unique berth number. Next, data points where the navigational status is not set to 5 are removed. For each event, the median longitude and latitude are calculated to determine the center coordinates of the berths, which are then passed to the DBSCAN algorithm with ϵ=50 m and $p_{n}=3$ for clustering purposes. Finally, convex hull polygons are created for each cluster. For post-processing, we incorporate an additional step by turning the clusters into rotated rectangles. This addition enhances the outcome, as can be seen in Figure 7.

The results of our proposed method, both with and without geohash encoding, as well as the competing method of Steenari et al. can be found in Table 2. The method by Steenari et al. scores (Mean: 13.780, SD: 0.507) consistently worse than the proposed method with (Mean: 0.836, SD: 0.097) and without geohash encoding (Mean: 1.008, SD: 0.132). This difference in performance is also visually evident in Figure 7, where the predictions from our proposed method, especially with geohash encoding, resemble more realistic berthing site configurations, producing coherent and practical berth placements. Moving on to a comparison between our two variants, Figure 7 highlights that the geohash-enabled variant produces more plausible outcomes than the non-geohash variant. For instance, the geohash-enabled approach avoids the unlikely scenario of identifying nine berths on the right side of the port, which is seen in the non-geohash variant. This improvement in realism demonstrates the effectiveness of incorporating geohash encoding, leading to more accurate and applicable port berth localization. Importantly, these qualitative improvements are consistent with the quantitative results, further reinforcing the superiority of the geohash-enabled approach. Furthermore, under identical settings—100 trials with fixed DBSCAN hyperparameters and GMM n_components capped (50 for small ports, 230 for large)—the geohash pipeline reduces average end-to-end runtime from 4.93 to 2.02 min across the 11 ports (mean speedup 1.76×; median 1.57×; range 1.05–3.09×).

**Figure 8:**
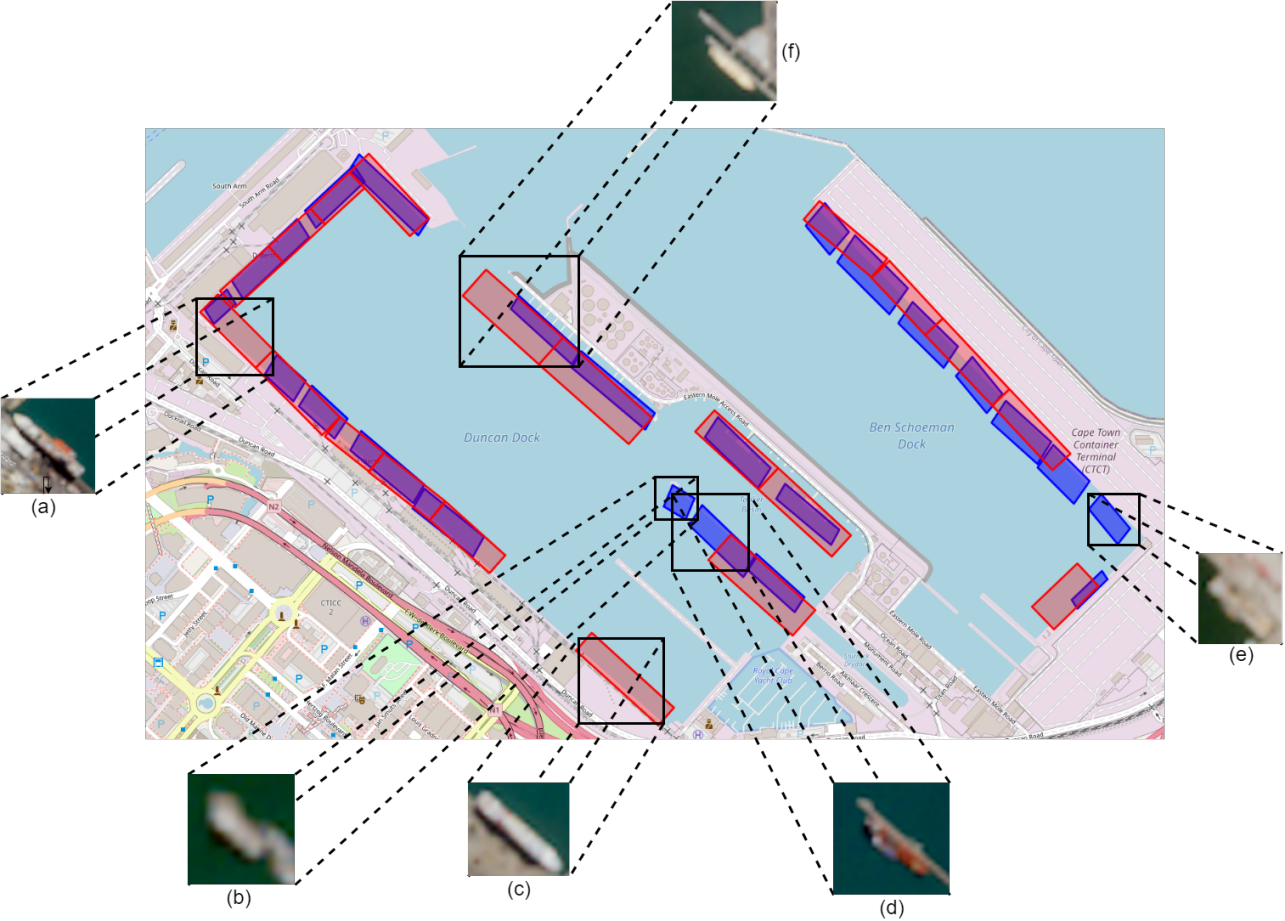
Qualitative results—Port of Cape Town. ShipNext berth labels (red) and model predictions (blue) with satellite insets: (**a**,**c**) sites missed by our model; (**b**,**d**,**e**) berths detected by the model but absent in ShipNext; (**f**) example where the model provides a more accurate berth outline.

Figure 8 presents a qualitative comparison between our method and the ShipNext’s labels. The depicted satellite images were identified from the Sentinel-2 optical archive by starting at the most recent (cloud-free) images and moving backwards in time (up to a year prior or until a ship was found at the region of interest). Our model demonstrates superior performance in several instances, identifying berths that are either omitted from ShipNext’s labels (b, d, e) or inadequately delineated (f). However, in two cases (a, c), our model fails to detect berths that are included in ShipNext’s labels. Upon investigation of these discrepancies, we find that no cargo or tanker emitted AIS data from the (a) and (c) regions for the POI. Additional investigation reveals that berth (a) is predominantly used by passenger/cruise ships and fishing vessels (ship types outside the scope of our investigation), while berth (c) serves mining vessels. Although mining vessels typically fall under the cargo category, the utilization of berth (c) appears to be either seasonal or to have only commenced after the POI (as confirmed by newer AIS data that indeed document mining vessel activity at this berth).

### 3.2. Ablations

We report on a number of ablations studies carried out to examine the importance of different components and characteristics of our data and method. For brevity, this section focuses on findings for the geohash-enabled method, which demonstrated consistently superior performance, while results for the non-geohash version are included in Appendix C.

#### 3.2.1. Period of Interest

**Figure 9:**
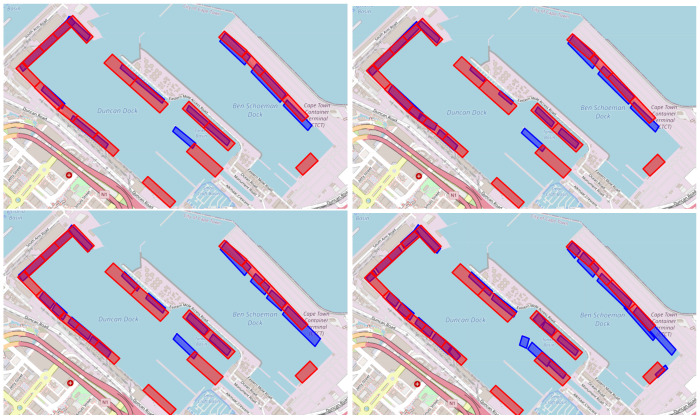
Effect of observation period (geohash variant). Predicted berths for Cape Town using Automatic Identification System (AIS) data windows of 3 days (BD: 1.617), 1 week (BD: 0.968), 2 weeks (BD: 1.162), and 1 month (BD: 0.830), displayed row-wise, left–to–right.

As shown in Table 3, extending the POI duration improves the consistency of GMM outputs across the two data splits. A qualitative comparison further supporting this observation is presented in Figure 9. This outcome aligns with intuition: longer observation windows yield more AIS messages, which allows for stricter DBSCAN hyperparameters without excessively pruning the dataset. As a result, both false positives (non-berth areas identified as berths) and false negatives (actual berths not detected) are less likely, and berth boundaries are more clearly delineated. These findings underscore the importance of using sufficient data to ensure robustness in berth localization.

#### 3.2.2. Number of Points Generated During Spatial Augmentation

This ablation study examines how varying the number of generated points per AIS message during spatial augmentation affects berth boundary precision and clustering consistency. We evaluated configurations with 0, 2, 5, 10, 20, and 40 additional points per message. Table 4 presents the corresponding BD for the port of Cape Town.

**Figure 10:**
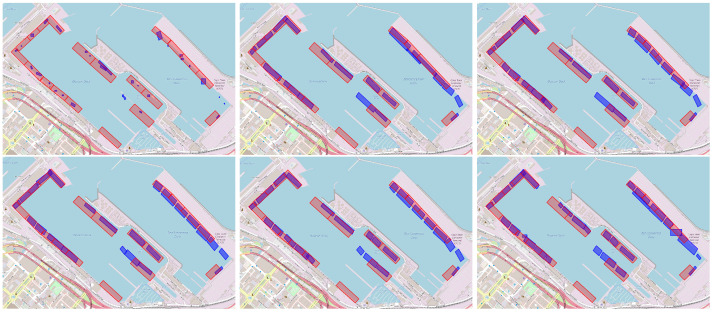
Effect of point generation (geohash variant). Results for Cape Town when generating 0 (BD: 1.23), 2 (BD: 0.66), 5 (BD: 0.65), 10 (BD: 0.64), 20 (BD: 0.63), and 40 (BD: 0.62) synthetic points per Automatic Identification System (AIS) message (displayed left–to–right, top–to–bottom).

Notably, when no additional points are generated (0 points), the BD is significantly higher, indicating poorer clustering consistency and underscoring the effectiveness of the proposed spatial augmentation. Qualitative results in Figure 10 corroborate this observation.

Moreover, Table 4 suggests that increasing the number of generated points generally improves clustering consistency. However, the incremental gains diminish as we move toward higher point counts. While adding points beyond 10 or 20 continues to yield marginal improvements, these may not justify the associated computational costs. The qualitative comparison in Figure 10 further supports the above conclusion, as minimal differences are observed at higher point densities. Based on these insights, we chose to generate 10 points during training and 20 points during evaluation.

#### 3.2.3. Interpolation Period

**Figure A4:**
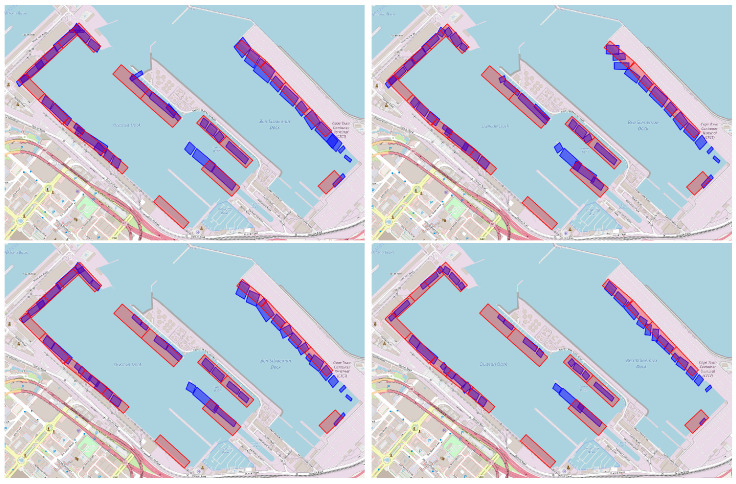
Effect of interpolation period (non-geohash variant). Qualitative results for Cape Town using interpolation intervals of 15 min (BD: 0.82), 30 min (BD: 0.83), 1 h (BD: 0.84), and 2 h (BD: 0.85) (displayed left–to–right, top–to–bottom).

This ablation study evaluates the sensitivity of the proposed method to varying interpolation periods in data preprocessing. Table 4 shows that 15 min, 30 min, and 1 h intervals yield comparable BD. However, shorter intervals (15 and 30 min) produce qualitatively unnatural berth delineations (see Figure A4), overly fragmenting the coastline into too many berths. Increasing to 1 h preserves stable delineations without unnecessary complexity, balancing both performance and computational cost. Extending to 2 h reduces computational cost further but introduces a mild performance decline, as seen in Table 4. Hence, the 1 h interpolation interval was chosen.

## 4. Discussion

In this work, we presented an unsupervised framework for port berth localization, evaluating two variants—one employing geohash encoding and one without—across a diverse set of 11 ports worldwide. Our results demonstrate a significant advancement over existing approaches in both quantitative and qualitative terms. By comparing our model predictions against satellite imagery and existing berth labels, we highlighted the limitations and inconsistencies in the current documentation. As presented, our models were able to identify berths that were overlooked or inaccurately represented by publicly available labels. Furthermore, the broad evaluation, encompassing ports of varying sizes and operational contexts, attests to the adaptability and generalizability of our approach.

A key factor in the success of our method lies in several novel methodological choices. First, the introduction of a spatial data augmentation strategy, guided by vessel dimensions and headings, enriched the model’s spatial representation and contributed to more coherent berth delineations. Second, our use of a KLD-based score, coupled with Bayesian optimization and the Minimum Description Length (MDL) principle, facilitated a principled and data-driven approach to hyperparameter tuning and model selection. Finally, the post-processing procedure, which transforms the probabilistic outputs of GMMs into polygonal berth boundaries, enabled a more intuitive and practical interpretation of the results. Each of these components, when combined, provided a robust and flexible framework capable of delivering reliable berth localization results across diverse maritime environments.

Our ablation studies further elucidated the factors influencing berth localization performance. Increasing the observation period (e.g., from a few days to one month) consistently yielded more stable and accurate GMM outputs, confirming the intuitive notion that more AIS messages provide a firmer statistical basis for identifying berths. Likewise, introducing spatial augmentation significantly enhanced clustering consistency, though the marginal gains diminished as the number of generated points increased, guiding us toward a balanced configuration. Setting the interpolation interval to one hour provided balance between computational efficiency and performance. Notably, these adjustments benefited the geohash-enabled variant more consistently than its non-geohash counterpart, underscoring the advantages of encoding spatial information into geohashes. While non-geohash models still improved through augmentation and tuning, their gains were not as pronounced, reinforcing the conclusion that geohash encoding provides a more robust and reliable foundation for data-driven berth localization. Beyond accuracy, the geohash pipeline is more computationally efficient, reducing average end-to-end runtime from 4.93 to 2.02 min across the 11 ports (mean speedup 1.76×; median 1.57×; range 1.05–3.09×).

Beyond the empirical gains we reported, our work offers three concrete strengths that we believe the pattern recognition community can directly benefit from: (i) a fully unsupervised, end-to-end pipeline whose hyperparameters are selected by internal, information-theoretic criteria (KL/MDL) rather than labels—making it deployable at any port with sufficient AIS data; (ii) a hierarchical, two-stage design (ship-level DBSCAN followed by area-level GMM) that explicitly separates denoising/episode detection from berth inference, which others can reuse in similarly noisy spatial–temporal problems; and (iii) a distributional, model–model validation protocol (BD/KLD) that can act as a general recipe for evaluating clustering stability when ground truth is unavailable.

Despite these advances, some limitations remain, offering fertile ground for future investigation. Currently, our approach focuses primarily on cargo and tanker vessels; extending coverage to other vessel types, such as passenger or fishing ships, could broaden the applicability of our method. While the geohash-enabled variant consistently demonstrated superior performance, some of our methodological choices and hyperparameter configurations (e.g., interpolation period) were designed to be generally effective across a diverse set of ports, potentially biasing the outcome toward a “one-size-fits-all” solution. Future work could tailor these configurations specifically to each variant and port, potentially revealing scenarios where the non-geohash variant might excel or reducing the need for certain preprocessing steps. Additionally, while our method leverages freely available terrestrial AIS data, regions with sparse coverage or data quality issues (e.g., Port of Ambarli) may pose challenges to the application of our method.

By bridging the gap between raw AIS data and port berth localization, this work provides a strong foundation for more informed decision-making within maritime logistics and port management. As global data accessibility continues to improve, our framework’s adaptability and scalability position it as a valuable tool for stakeholders aiming to optimize port operations. Ultimately, advances in unsupervised berth localization can pave the way for more agile, data-driven maritime strategies and more resilient global trade networks.

### Future Directions

Future research directions aimed at enhancing the method’s effectiveness and reliability include (i) extending the POI to multi-month/seasonal windows to capture intermittently used berths; (ii) incorporating multi-modal weak supervision (Sentinel-2/SAR imagery and third-party berth polygons) for semi/weakly supervised outline refinement; (iii) broadening evaluation across additional ports and vessel classes; and (iv) developing task-specific variants, e.g., ship-type/subtype–specific models and hotspot mapping beyond ports.

## Figures and Tables

**Table 1:** Selected ports with their respective annual twenty-foot equivalent units (TEUs) and the number of AIS messages received from AISStream over a period of 1 month.

Port	Country	TEU (k)	AIS Messages	Port	Country	TEU (k)	AIS Messages
Singapore	Singapore	37,000	612,406	Auckland	New Zealand	818	22,814
Busan	South Korea	22,000	195,627	Livorno	Italy	800	56,284
Antwerp	Belgium	13,000	394,162	Cape Town	South Africa	720	40,088
Los Angeles	USA	5000	41,462	Gdansk	Poland	685	67,492
Ambarli	Turkey	2600	13	Limassol	Cyprus	348	15,631
Southampton	UK	1600	29,290	Algeciras	Spain	337	27,386

**Table 2:** BD-based comparison of our method and that of Steenari et al. on AIS data of one month, averaged over 200 runs.

	Non-Geohash		Geohash		Steenari et al. [26]	
**Port**	**Mean**	**SD**	**Mean**	**SD**	**Mean**	**SD**
**Singapore**	0.882	0.057	**0.850**	0.042	9.979	0.191
**Busan**	0.800	0.143	**0.687**	0.135	14.870	0.776
**Antwerp**	1.145	0.071	**0.957**	0.057	10.383	0.133
**Los Angeles**	0.950	0.046	**0.855**	0.035	*∞*	*∞*
**Southampton**	0.985	0.199	**0.672**	0.132	16.887	0.680
**Auckland**	1.237	0.258	**0.922**	0.193	12.487	0.437
**Livorno**	1.020	0.340	**0.830**	0.236	15.458	0.507
**Cape Town**	0.815	0.082	**0.618**	0.066	16.751	0.511
**Gdansk**	1.235	0.084	**0.955**	0.057	14.736	0.817
**Limassol**	0.734	0.080	**0.705**	0.060	13.192	0.501
**Algeciras**	1.288	0.088	**1.142**	0.053	13.058	0.518
**Average**	1.008	0.132	**0.836**	0.097	13.780	0.507

**Table 3:** Ablation study results for the geohash-enabled method showing the effect of the POI on performance (BD). Left and right columns represent different sets of ports.

Port	3 Days	1 Week	2 Weeks	1 Month	Port	3 Days	1 Week	2 Weeks	1 Month
					**Gdansk**	3.064	1.611	1.203	**0.922**
**Algeciras**	3.615	2.046	1.475	**1.142**	**Limassol**	1.137	0.940	0.763	**0.672**
**Antwerp**	1.838	1.381	1.169	**0.705**	**Livorno**	1.326	1.076	1.065	**0.855**
**Auckland**	2.456	1.157	1.135	**0.955**	**Los Angeles**	6.791	1.521	1.060	**0.957**
**Busan**	1.246	0.959	0.790	**0.618**	**Singapore**	1.303	1.039	0.878	**0.687**
**Cape Town**	1.617	0.968	1.162	**0.830**	**Southampton**	2.020	1.105	0.877	**0.850**

**Table 4:** Ablation study results for the Port of Cape Town. Left: Impact of spatial augmentation (number of generated points) on geohash-based methods. Right: Sensitivity to different interpolation periods. Performance is measured using the BD. L-CI and U-CI stand for lower and upper confidence interval bounds (95%), respectively.

Generated Points	Mean	L-CI	U-CI	SD	Interpolation	Mean	L-CI	U-CI	SD
**0 points**	1.230	1.167	1.294	0.645					
**2 points**	0.657	0.650	0.665	0.077	**15 min**	0.621	0.612	0.630	0.067
**5 points**	0.654	0.644	0.664	0.072	**30 min**	0.616	0.606	0.626	0.069
**10 points**	0.641	0.631	0.650	0.068	**1 h**	0.619	0.610	0.629	0.069
**20 points**	0.625	0.615	0.634	0.067	**2 h**	0.632	0.623	0.641	0.067
**40 points**	0.621	0.615	0.627	0.065					

**Figure A1:**
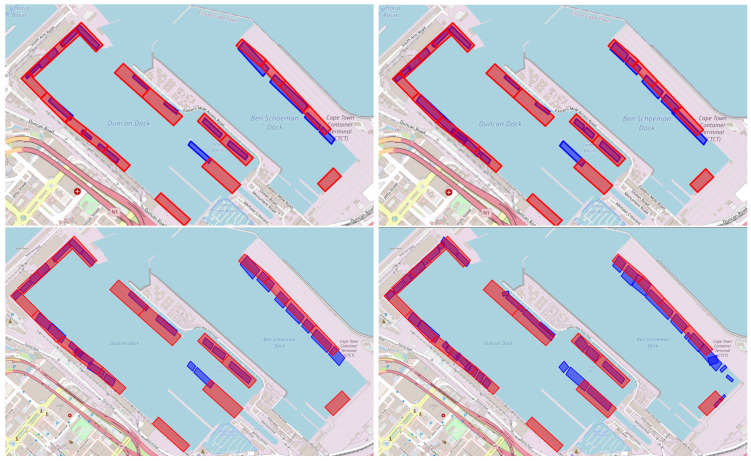
Effect of observation period (non-geohash variant). Predicted berths for Cape Town using Automatic Identification System (AIS) data windows of 3 days (BD: 2.16), 1 week (BD: 1.39), 2 weeks (BD: 1.33), and 1 month (BD: 0.99) (displayed left–to–right, top–to–bottom).

**Figure A2:**
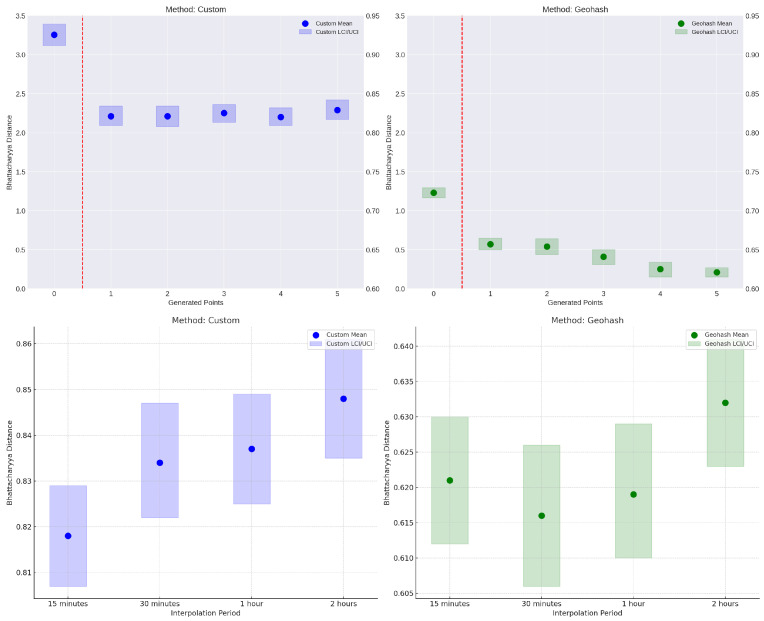
Metrics for the generation of points and interpolation periods ablations. BD for the point-generation ablation (**left**) and the observation-period ablation (**right**); the red line separate the no point augmentation case which requires a different y-axis range.

**Figure A3:**
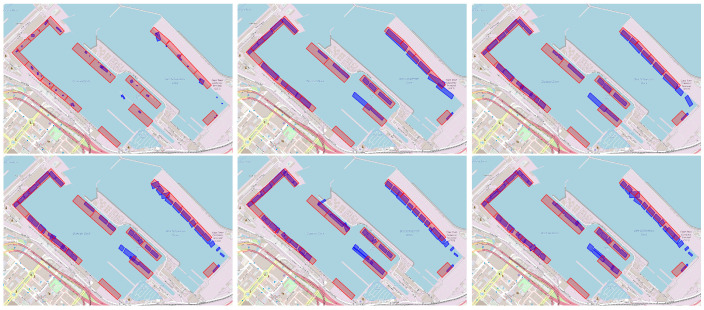
Effect of point generation (non-geohash variant). Cape Town results when generating 0 (BD: 3.25), 2 (BD: 0.82), 5 (BD: 0.82), 10 (BD: 0.83), 20 (BD: 0.82), and 40 (BD: 0.83) synthetic points per Automatic Identification System (AIS) message (displayed left–to–right, top–to–bottom).

## Data Availability

Research data for this article are made available via Mendeley Data (DOI: 10.17632/r37vwd493d.1) at https://data.mendeley.com/datasets/r37vwd493d/1 (accessed on 4 November 2025).

## Abbreviations

The following abbreviations are used in this manuscript:

MDL	Minimum Description Length
GMM	Gaussian Mixture Model
DBSCAN	Density-based spatial clustering of applications with noise
KLD	Kullback-Leibler divergence
TEU	twenty-foot equivalent unit
SD	Standard deviation
MCI	Monte Carlo Integration
API	Application Programming Interface
POI	Period of Interest
ROI	Region of Interest
MMSI	Maritime Mobile Service Identity
TPE	Tree-structured Parzen Estimator
BD	Bhattacharyya distance
PDF	Probability density function

## Appendix A. Data Preprocessing

Table A1 details the number of AIS messages after each prepossessing step over the POI and across all ports under investigation. Initially, the data are refined by selecting only records with a speed below a maximum threshold. Next, AIS messages with missing dimension information are excluded—however, all AIS messages in our experiments were complete in that sense. Records with a heading of “511”, indicating unavailable heading data, are then discarded. The data are then divided into two approximately equal parts (as described in the main text). In the interpolation step, data for each vessel is interpolated on an hourly basis. Finally, any records with significant heading changes (herein defined as larger than 10 degrees) are removed from these splits.

**Table A1:** One-month period AIS message count following each preprocessing step.

Port	AIS Data	ROI	Speed	511 Heading	Split		Interpolation		Heading	
**Singapore**	609,975	119,466	103,624	95,369	47,870	47,499	25,561	25,414	23,559	23,483
**Busan**	120,752	90,363	76,037	54,491	27,477	27,014	14,753	14,712	10,636	10,124
**Antwerp**	676,896	322,840	281,909	104,479	52,514	51,965	27,950	27,666	25,723	25,539
**Los Angeles**	42,055	11,249	10,954	10,954	5575	5379	2822	2743	2758	2684
**Southampton**	24,998	8383	7382	7243	3905	3338	2031	1746	1872	1651
**Auckland**	5618	5587	5587	5184	2919	2265	1484	1174	1420	1115
**Livorno**	19,700	14,476	13,382	13,127	6690	6437	3455	3313	3304	3148
**Cape Town**	17,863	11,030	10,444	10,398	5398	5000	2757	2535	2655	2470
**Gdansk**	72,020	38,192	37,311	32,314	16,480	15,834	8404	8079	8230	7910
**Limassol**	10,049	2131	1899	1690	874	816	464	441	404	402
**Algeciras**	76,008	15,368	12,335	11,971	6099	5872	4173	3957	3058	2714

## Appendix B. Optimal Hyperparameter Values

The optimal hyperparameter values for both DBSCAN and GMM, as determined through our model selection process, are presented in Table A2 for all ports of interest, encompassing both the geohash and non-geohash variants of the proposed method.

**Table A2:** Optimal hyperparameters for both the Geohash and Non-Geohash methods over a one-month period.

Period: 1 Month	Non-Geohash			Geohash		
**Port**	**Epsilon (m)**	**MinPoints**	**nComponents**	**Epsilon (m)**	**MinPoints**	**nComponents**
**Algeciras**	33.509	2	49	35.215	2	49
**Auckland**	37.445	2	27	34.841	2	21
**Antwerp**	14.630	2	228	53.296	3	228
**Busan**	34.687	2	49	5.630	7	48
**Cape Town**	55.004	9	42	35.454	13	23
**Gdansk**	52.742	5	49	22.829	4	48
**Limassol**	17.319	3	10	37.060	2	10
**Livorno**	27.159	2	49	15.778	5	41
**Los Angeles**	19.797	5	49	42.313	14	40
**Singapore**	49.436	2	228	35.830	2	226
**Southampton**	26.180	5	48	12.750	4	32

## Appendix C. Further Ablation Results

### Appendix C.1. Period of Interest

As shown in Table A3, increasing the POI duration generally improves GMM consistency across the two data splits, although exceptions exist. Notably, in Limassol and Southampton, the 2-week interval slightly outperforms the 1-month interval, with statistical significance only at Southampton. A qualitative comparison for the Port of Cape Town (Figure A1) reveals that while shorter durations (3 days and 1 week) result in sparse berth localization, the difference between 2 weeks and 1 month is less pronounced than in the geohash variant. Nevertheless, given the overall trend and performance, choosing the 1-month POI remains the more favorable option in the non-geohash setting.

**Table A3:** Ablation study results of the non-geohash variant across the ports showing the effect of the POI on performance (BD). Left and right columns represent different sets of ports.

Port	3 Days	1 Week	2 Weeks	1 Month	Port	3 Days	1 Week	2 Weeks	1 Month
					Gdansk	3.649	2.121	1.656	**1.237**
Algeciras	3.677	2.302	1.661	**0.882**	Limassol	1.277	1.135	**0.939**	1.020
Antwerp	2.312	1.738	1.462	**0.800**	Livorno	1.786	1.467	1.444	**0.815**
Auckland	1.944	1.492	1.547	**1.145**	Los Angeles	6.885	1.821	1.236	**1.235**
Busan	1.530	1.022	0.954	**0.950**	Singapore	1.494	1.184	0.981	**0.734**
Cape Town	2.160	1.386	1.329	**0.985**	Southampton	2.369	1.504	**1.097**	1.288

### Appendix C.2. Number of Points Generated During Spatial Augmentation

As shown in Table A4, introducing any number of additional points substantially improves clustering consistency compared to having no additional points, confirming the effectiveness of spatial augmentation. However, unlike the geohash-enabled variant, increasing the number of generated points beyond a minimal threshold does not yield clear incremental gains (see Figure A2). Qualitative results in Figure A3 further support this: higher point counts neither improve berth delineations nor provide clearer boundaries, and instead risk identifying too many berths (overfitting). Given the lack of quantitative evidence to favor a different approach, we adopt the same configuration as in the geohash variant for consistency.

**Table A4:** Ablation study results for the Port of Cape Town, illustrating (first two columns) the impact of spatial augmentation and the sensitivity to varying numbers of generated points, and (last two columns) the impact of different interpolation times in the proposed non-geohash method. Performance is measured using the BD.

Generated Points	Mean BD	Interpolation Period	Mean BD
**0 points**	3.254		
**2 points**	0.821	**15 min**	0.818
**5 points**	0.821	**30 min**	0.834
**10 points**	0.825	**1 h**	0.837
**20 points**	0.820	**2 h**	0.848
**40 points**	0.829		

### Appendix C.3. Interpolation Period

As shown in Table 4, shorter intervals (notably 15 min) again achieve slightly better quantitative results, but at the cost of increased computational complexity. Unlike the geohash variant, which exhibited unnatural berth delineations at very short intervals, the non-geohash variant does not present such clear qualitative drawbacks (see Figure A4), making it difficult to identify a definitive winner. Nevertheless, these differences are not substantial enough to justify adopting a separate configuration from the geohash-based method. Given the acceptable performance at 1 h and the associated efficiency benefits, we maintain the same 1-h interpolation period for both variants. This ensures consistency across experiments while balancing performance and computational cost.
